# A Simple and Highly Specific MassARRAY-Based Stool DNA Assay to Prioritize Follow-up Decisions in Fecal Immunochemical Test-Positive Individuals

**DOI:** 10.3390/cancers11030423

**Published:** 2019-03-25

**Authors:** Pi-Yueh Chang, Chia-Chun Chen, Jy-Ming Chiang, Shih-Cheng Chang, Mei-Chia Wang, Jinn-Shiun Chen, Wen-Sy Tsai, Jeng Fu You, Jang-Jih Lu

**Affiliations:** 1 Department of Laboratory Medicine, Chang Gung Memorial Hospital at LinKou Taoyuan 33305, Taiwan; changpy@cgmh.org.tw or changpy@adm.cgmh.org.tw (P.-Y.C.); changsc137@cgmh.org.tw (S.-C.C.); ottermika@cgmh.org.tw (M.-C.W.); 2 Department of Medical Biotechnology and Laboratory Science, Chang Gung University, Taoyuan 33302, Taiwan; 3 Molecular Medicine Research Center, Chang Gung University, Taoyuan 33305, Taiwan; chenchiachun@gap.cgu.edu.tw; 4 Department of Colorectal Surgery, Chang Gung Memorial Hospital at Linkou, Taoyuan 33305, Taiwan; jmjiang@cgmh.org.tw (J.-M.C.); chenjs@cgmh.org.tw (J.-S.C.); wensyt@gmail.com (W.-S.T.); you3368@cgmh.org.tw (J.F.Y.)

**Keywords:** colorectal cancer, stool DNA, MassARRAY, risk-stratifying algorithm

## Abstract

Background: Seventy-five percent of fecal immunochemical test (FIT)-positive individuals are false positives and undergo unnecessary colonoscopies. Here, we established a stool DNA (sDNA) test that uses the Single Allele Base Extension Reaction (SABER) MassARRAY platform to improve the accuracy of FIT-based CRC detection. Methods: Twenty-one variants in five CRC-associated genes were selected for the sDNA panel. Cell line DNA and matched mutation-confirmed tissue and stool samples from 34 patients were used for accuracy assessment (cohort 1). The clinical performance of the sDNA assay was further evaluated in 101 independent FIT-positive stool samples (cohort 2). Results: In cohort 1, we obtained a 62% mutation concordance rate in paired tissue and stool samples of the CRC group, regardless of the FIT status. In cohort 2, 100% specificity in normal controls with positive FIT results was observed. By weighting the FIT value and the presence of a given variant type in stool and then summing the two scores, we found that a one-increment increase in the score was associated with a 4.538-fold risk (95% CI = 2.121–9.309) for malignancy in the FIT-positive setting. Conclusions: Our highly specific sDNA assay can help prioritize the most at-risk FIT-positive persons to receive prompt colonoscopic confirmation of CRC.

## 1. Introduction

Colorectal cancer (CRC) is the most common cancer that affects both men and women. The globally recognized CRC screening test is the fecal immunochemical test (FIT), which detects the presence of blood in feces. However, FIT is not entirely reliable: it has a positive predictive value of only 5% for CRC and 20% for advanced polyps in FIT-positive individuals [1], and 75% of FIT-positive individuals have false positive results due to hemorrhoids or harmless small polyps. Meanwhile, FIT-positive individuals are usually referred for colonoscopy for visualization of possible lesions throughout the intestinal lumen [2]. Since 2004, the Taiwanese government has provided free biannual FIT for those 50–75 years of age. At 12 years after the inauguration of this program, 56.6% of eligible individuals had actively participated, and a 44% reduction in CRC-related mortality had been observed [3]. The implemented FIT screening strategy has thus enhanced public health. However, the need for colonoscopic confirmation in the FIT-positive group, which has represented 8% of screened individuals on average, has strained both finances and health workers [3]. There is thus an unmet need for more specific supplementary tests that can help prioritize FIT-positive individuals with respect to CRC risk.

Biomarkers are deposited in stool by active secretion or leakage from neoplasms or via exfoliation of colonic epithelial cells. Various carcinogenesis-related alterations can be detected in stool, such as somatic mutations in *APC*, *KRAS*, and *TP53*; microsatellite instability in *BAT-26*; methylation of markers in *SEPT9* and *SFRP1* [4]; and DNA integrity [5]. Studies have shown that stool DNA (sDNA) tests for the above-listed changes have sensitivities of 51.6% to 100% for CRC and 10.5% to 81.8% for adenoma, with specificities around 95% [6]. However, the above-listed sDNA tests can be challenged by the relative lack of human DNA amidst the food residue and microorganisms that comprise stool (<0.1% of the total DNA, the rest of which corresponds to bacterial DNA). The sDNA tests reported to date have thus required the collection of large stool samples and/or the addition of anti-DNA degradation buffers.

In 2014, FDA approved Cologuard^TM^ as a screening tool for average-risk populations. This kit examines two DNA methylation markers (*NDRG4* and BMP3), seven *KRAS* mutation markers, one beta actin normalization marker, and one conventional fecal hemoglobin marker to get a high sensitivity of 92.3% for CRC and 42.4% for advanced adenoma, with a specificity of 86.6% in 9,989 asymptomatic individuals [7]. Although Cologuard^TM^ exhibits encouraging performance, large amounts of stool are required and an exclusive central lab is needed to handle the sophisticated and expensive testing and analysis. This limits the feasibility of using Cologuard^TM^ a first-line routine test. Moreover, researchers subsequently found that Cologuard^TM^ false-positive individuals had the same cumulative cancer incidence as the general population after long-term follow-up [8]. Thus, medical care resources may have been wasted due to the relatively low specificity of the panel. Regarding the literature, it is notable that most of the existing studies on sDNA assays have examined the detection rate in stool samples of different subgroups (e.g., adenoma or CRC groups), whereas few have compared the DNA alterations found in stool samples and their matched tissue samples [9]. This is an important component that should be addressed when considering the validity and reliability of an sDNA assay.

To address the existing need for a more specific assay method and address this gap in the literature, we herein sought to construct an sDNA assay that is simple enough for routine implementation and can be used to prioritize FIT-positive individuals for further colonoscopic assessment. We wanted to develop a platform that: (1) uses a simple commercial kit to extract DNA from a small amount of stool; (2) assesses gene variants that offer maximum coverage for the presence of early neoplasm and exclude other non-relevant disorders; and (3) uses a detection platform that is as sensitive as possible while remaining easy to apply. To check the accuracy of our developed method, we collected 34 stool samples from patients with known tissue mutations that had been identified by next-generation sequencing (NGS) in our previous studies [10,11]. The clinical performance of our sDNA test was validated in 101 independent FIT-positive samples. Finally, we improved the potential practical application of our method by establishing a risk-stratifying algorithm.

## 2. Results

### 2.1. Accuracy and Analytical Sensitivity of SABER MassARRAY Assay, as Evaluated in LoVo Cell Line and Tissue DNA

**Figure 1:**
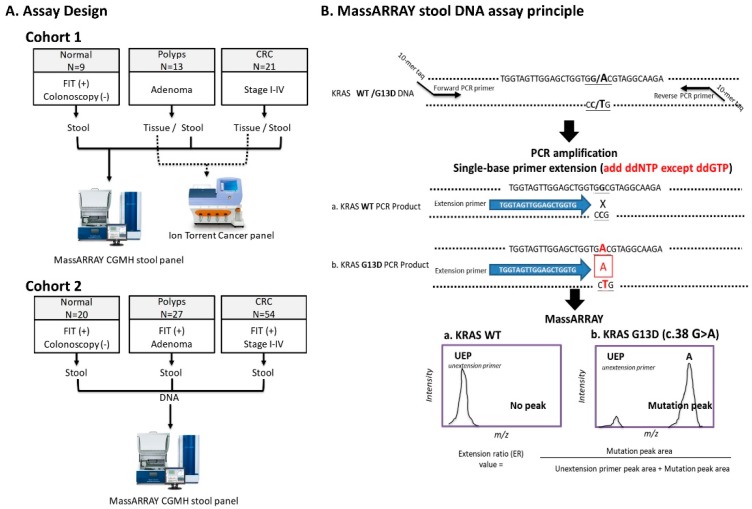
Assay design and the detection principles of the MassARRAY stool DNA panel. (**A**) Two cohorts that included normal, polyp-bearing, and CRC patients with available FIT data and colonoscopy results were separately used to evaluate the accuracy and clinical performance of the developed stool DNA (sDNA) assay. DNA from tissue samples with known DNA with mutation profiles, as previously assessed using the Cancer Hotspot Panel on Ion Personal Genome Machine, were collected, along with the corresponding paired pre-operational stool samples, from normal, polyp and CRC patients in cohort 1. Cohort 2 stool samples were collected from a FIT-positive population. All stool mutations were detected using the Sequenome MassARRAY platform. (**B**) Illustration of the principles and procedures of the single allele base extension reaction (SABER), using *KRAS* p.G13D as an example. UEP: unextension primer peak; *m*/*z*: mass-to-charge ratio; ER: extension ratio.

**Figure 2:**
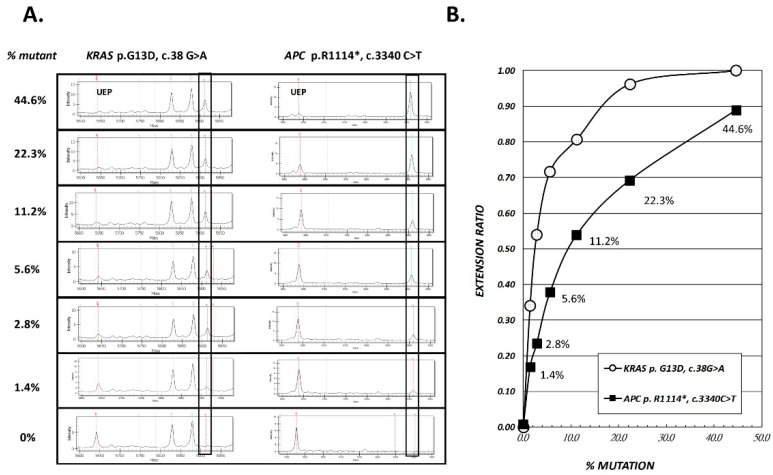
Accuracy and analytical sensitivity check of sDNA panel using the LoVo cell line. Two mutations, KRAS p.G13D and APC p.R1114*, are known to be harbored in the LoVo cell line and could be detected by the sDNA panel. (**A**) Mass spectrometric (MS) analysis of two mutations, with their mutant percentages shown. The marked rectangular boxes indicate the expected size of each mutant extension product. (**B**) Regression curves of the calculated extension ratio (ER) and mutation % values for the two mutants.

The sDNA panel consists of 21 variants in the five most frequently mutated genes in CRC and polyp tissues of our Taiwanese patients (Table 1). The principle of Single Allele Base Extension Reaction (SABER) MassARRAY method and assay design are depicted in Figure 1 and Table 2 (details see Materials and Methods). First, we evaluated the accuracy of our MassARRAY platform in DNA from the LoVo cell line (which harbors two of the screened mutations) and 11 tissue samples with one or two previously identified variants. The cell line DNA was serially diluted with wild-type DNA (1:1 ratio) to yield samples containing 44.6% to 1.4% of the variant DNA. The specific extension products corresponding to *KRAS*_c.38G > A and *APC*_c.3340C > T were successfully detected by MassARRAY, corresponding to specific peaks at 5912.9 Da and 6909.6 Da, respectively (Figure 2A). The extension ratios (ER) of the specific spots were positively correlated with the estimated allelic frequencies. The ER of the variants at 1.4% of dilution points was greater than the zero point and could easily be differentiated from the background signal (0.17 for *APC*_c.3340C > T and 0.34 for *KRAS*_c.38G > A vs. 0 for the background). Therefore, the analytic sensitivity was at least as low as 1.4% (Figure 2B) for the two tested variants. The other 16 variants were also detected as clearly distinguishable peaks at their expected positions, with similarly low detection limits of around 2% (Supplementary Materials Figure S1).

### 2.2. Performance of MassARRAY Stool DNA Panel in Cohort 1 Samples and Concordance of Mutations Detected in Stool and Matched Tissue Samples

**Figure 3:**
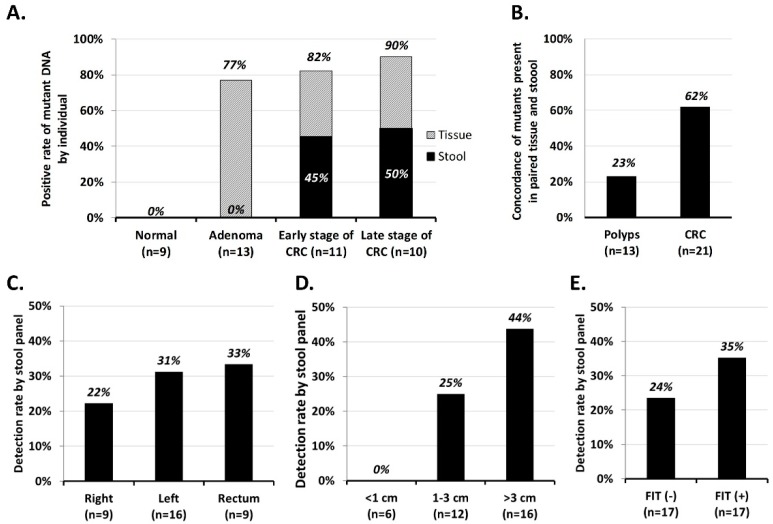
Correlation of the mutant detection rate of the sDNA test and various clinicopathological factors in cohort 1.

To assess the reliability of the MassARRAY stool DNA panel, we selected archived stool samples from 34 patients (21 with CRC and 13 with polyps, all having known tissue mutation profiles) and nine healthy controls, and grouped the samples as cohort 1. Since the mutant DNA in stool is theoretically derived from the lesion(s) in the colon, we expected that the mutations identified in stool would correspond to those in the tissue sample. Therefore, we carefully selected 28 stool samples with 19 of the variants that can be detected by the sDNA panel (examples were not included for KRAS p.G12C or PIK3CA p.T1025A). Mutation concordance was defined by the presence or absence of the same mutation profile in paired tissue and stool samples (Table 3). All stool samples passed the albumin internal control evaluation and no inhibitor issue was found in the stool DNAs. No mutation was found in the nine control stool samples; the ER values of the 21 target variants in these control samples were used to calculate the threshold to be used for each variant in patient stool samples. Overall, the expected and measured mutation detection rates in stool were 0%, 77%, 82%, 90% and 0%, 0%, 45%, 50% in normal, adenoma, early-stage CRC, and late-stage CRC, respectively (Figure 3A). Further comparison revealed that the mutual mutation pattern in paired tissue and stool samples had a concordance rate of 23% (3/13) in polyps and 62% (13/21) in the CRC groups (Figure 3B). Among the 19 expected variants, eight were detected in this trial (p.R876* and p.R1114* in APC; p.G12V and p.G12C in KRAS; p.R175H and p.R213* in TP53; p.E545K and p.T1025A in PIK3CA). When we analyzed the expected detection rate for each individual gene, we found that the *SMAD4* mutation was not detected in stool samples. *PIK3CA* was the most sensitive target, exhibiting the highest detection rate in stool samples whose paired tissue samples had mutations in this gene (50%, 3/6). However, *KRAS* has the highest positive rate in the CRC group (19%, 4/21), followed by *PIK3CA* (14%, 3/21).

Figure 3C–E summarize our efforts to correlate the stool DNA detection rate with various clinical variables. We found that: (1) the stool mutant detection rate was lowest when the lesion was located on the right side of the colon and highest in the rectum (Figure 3C); (2) the detection rate paralleled the lesion size, with a detection rate of 0% in stool from patients with a lesion size < 1 cm, 25% in those with a lesion size of 1-3 cm, and 44% in those with a large neoplasm (Figure 3D); and (3) sDNA positivity was independent of the FIT status (*p* = 0.452). In 17 FIT-negative cases among the patients of this cohort, the stool samples of four were identified as having mutations in *PIK3CA* or *APC* (Figure 3E and Table 3).

### 2.3. Validating the Performance of the sDNA Panel in Cohort 2

**Figure 4:**
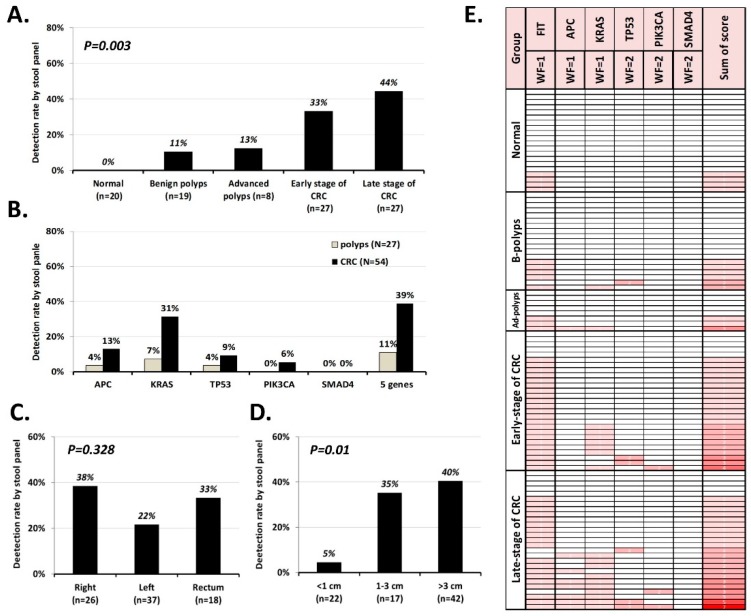
Clinical validation of the sDNA test in cohort 2. (**A**–**D**). Detection rate versus various clinicopathological factors and gene mutations. (**E**). A risk-stratified scoring system was constructed by integrating the FIT value (FIT ≥ 500 ng/mL, weighting factor = 1), the presence of mutations in *APC*, *KRAS* (weighting factor = 1 for either), *TP53*, *PIK3CA*, and *SMAD4* (weighting factor = 2 for each). The gradient color represents the score.

To further validate and understand the performance of our sDNA assay, we prospectively collected 101 FIT-positive stool samples as cohort 2. As illustrated in Figure 4A, the detection rate of stool mutant DNA increased from patients with benign polyps (11%) to those with late-stage CRC (44%), and 100% specificity was observed in the normal control group. Unlike the findings in cohort 1, the most prevalent mutations in cohort 2 were found in KRAS (Figure 4B). In total, 10 variants were detected in stool samples (p.R876*, p.R1114* and p.R1450* in APC; p.G12A, p.G12D, p.G12V, p.G13D and p.A146T in KRAS; p.R175H in TP53; p.E545K in PIK3CA).The mutation detection rate was independent of the lesion location (Figure 4C), but it was found to be affected by lesion size (Figure 4D).

The original goal for designing the sDNA panel was to enable FIT-positive individuals at high risk for CRC to be prioritized for prompt colonoscopy while reducing unnecessary procedures for lower-risk individuals. Therefore, the high specificity of the test is welcome. However, there is typically a trade-off between specificity and sensitivity, and the rather low sensitivity of the sDNA panel in detecting CRC and precursor adenoma could delay the observation of neoplasms. We speculated that since the quantitative FIT value and the number and type of mutations correlated well with disease severity, we might be able to generate a scoring system that incorporates the two kinds of information. Lee et al. [12] reported that CRC mortality was positively associated with the FIT concentration; patients with 100+ μg hemoglobin/g of feces had a 2.53-fold mortality rate compared to those with a normal level of hemoglobin (<20 μg) in their stool. The 100 μg hemoglobin level used by Lee et al. [13] is equivalent to 500 ng/mL in the present study. Our previous tissue genome studies revealed that alterations in *APC* and *KRAS* appeared earlier during the formation of adenoma, whereas mutations in *TP53*, *PIK3CA* and *SMAD4* were acquired for tumor transformation and play driver roles in tumor biology [11]. Therefore, to construct the risk-stratifying algorithm we gave a weight factor of 1 to a FIT value >500 ng/mL and the presence of a mutation in *APC* and/or *KRAS*, and a weight factor of 2 to the presence of a mutation in *TP53*, *PIK3CA*, and/or *SMAD4*. These weighted values were summed to obtain a score. Supplementary Materials Table S2 lists the FIT values, the variants detected by our MassARRAY sDNA panel, and the summed score of each of the 101 participants in cohort 2. 

Figure 4E presents the score distribution, which ranged from normal samples to those representing late-stage CRC, by gradient color. Samples with no mutation and FIT <500 ng/mL had a score of 0 (no color), while a single late-stage CRC sample with FIT ≥ 500 ng/mL and a total of five mutations across *APC*, *KRAS*, *TP53*, and *PIK3CA* had a total score of 7 (red color). If we simply stratified the FIT-positive cases as benign (normal + benign-adenoma) or malignant (advanced adenoma + all stages of CRC) based on their summed scores, the area under the receiver operating characteristic curve was 0.781 (95% confidence interval [CI] = 0.691–0.871, *p* < 0.001). Logistic regression after adjustment for age and gender showed that there was a 4.589-fold increase in the malignancy risk (95% CI = 2.228–9.450, *p* < 0.001) for each score increment in the FIT-positive population.

## 3. Discussion

The ability of FIT to identify as many high-risk individuals as possible at the initial screening phase can help reduce cancer mortality [14]. The average 67% of colonoscopy confirmation procedure after positive FIT results has limited the positive impact of this screening technique [3], but FIT-positive individuals who did not comply with colonoscopy were found to have a 1.64-fold increased risk of CRC death compared with those who complied [3]. This indicates the need for a tool that can help clinicians identify and prioritize individuals with the highest risk for developing malignancy, while allowing lower-risk individuals to take a wait-and-see approach that will reduce unnecessary procedures. In this study, we established a simple SABER MassARRAY-based stool DNA assay that incorporated the 21 most frequent CRC-related mutations of the COSMIC database that also showed a high incidence in the Taiwanese population. The average analytical sensitivity of most variants was 1–2%, as assessed in serially diluted cell line DNA and tissue DNA. With the exception of certain variants that proved relatively analytical insensitive (such as *APC* c. 4348 C > T and *APC* c. 4661dupA in Supplementary Materials Figure S1) or barely amounts of variants detected in stool, we observed 62% concordance of the mutations detected in tissues and their matched stool samples. The results obtained using stool samples from 101 independent FIT-positive individuals also indicated that our sDNA assay increased the accuracy of FIT with higher specificity. We also introduce a risk-stratifying algorithm that combines the results from our assay with the FIT value to generate a score that could potentially assist physicians in persuading noncompliant individuals to accept the invasive confirmation procedure.

As detection tools, sDNA tests have proven more reliable than FIT, since they reflect the genomic alterations seen during CRC development. However, the utilized markers and the obtained results have varied. Where most of the other stool panels have screened for only *KRAS* mutation [13,15], our stool DNA panel can achieve a higher detection rate by examining mutations in five genes involved in different stages of carcinogenesis. In the present study, 13 variants distributed in four genes were detected in the stool samples of patients with CRC or polyps (p.R876*, p.R1114* and p.R1450* in APC; p.G12S, p.G12A, p.G12D, p.G12V, p.G13D and p.A146T in KRAS; p.R213* and p.R175H in TP53; p.E545K and p.T1025A in PIK3CA). Of them, 54% (7 variants) were located in *APC, TP53*, and *PIK3CA*. Recently, Youssef et al. [16] used NGS to survey the mutation patterns in 87 CRC stool samples, and found 20 mutations in 11 genes. Of them, *APC* and *TP53* were the most frequently mutated genes. Those findings highlight the importance of examining multiple CRC-related variant genes instead of solely focusing on *KRAS*. 

The nature of sDNA testing overcomes the dilemma of obtaining a false-negative FIT result from a single non-bleeding stool sample. Theoretically, free DNA and that found in exfoliated apoptotic colonocytes will continuously shed into stool. Moreover, the genetic alterations observed in the stool were reported to be similar in FIT-positive and -negative CRC patients [9]. Therefore, it is not surprised that four of the 17 FIT-negative cases in cohort 1 had positive sDNA findings. Consistent with the previous proposal that the mutation pattern in stool should be more informative than that in the original lesion [17] due to tumor heterogeneity [18], one CRC stool sample of cohort 1 harbored a PIK3CA p.T1025A mutation that was not detected in the matched tissue. The discrepancy could reflect that multiple subclones with different mutations can arise and develop simultaneously [19], such that there are local differences in the mutation profile. In such cases, sDNA will be more likely to represent the overall mutational profile, making sDNA assays more reliable and sensitive than FIT.

In terms of clinical practice, a screening assay should involve a robust and feasible platform and be generally affordable. However, the only FDA-approved stool DNA kit, Cologuard^TM^, requires 36 g of stool and a lengthy, complicated sample processing procedure to accomplish the detection of nine biomarkers. Itzkowitz et al. [20] constructed a sDNA assay for detecting hypermethylation of the vimentin gene and a marker of DNA integrity, but the method required the immediate addition of DNA preservation buffer to fresh stool and gel-based purification of DNA. The method described in the present study requires only 200 mg of stool and a relatively simple DNA extraction procedure. Regarding platform selection, there are several highly sensitive technologies that can detect rare variants against an overwhelming wild-type background, such as peptide nucleic acid (PNA)-mediated clamping PCR, BEAMing, and digital melting-curve analysis [21,22]. Recently, various matrix-assisted laser desorption ionization-time of flight (MALDI-TOF)-based assays were approved as ultrasensitive mutation detection methods and evaluated for their ability to detect lung cancer-related EGFR mutations in cell-free DNA [23]. To our knowledge, however, this is the first study in which a MassARRAY platform has been used to detect CRC-related mutations in stool. We herein demonstrate a SABER MassARRAY-based method that is feasible for clinical application, with an analytical sensitivity of 1–2% and validated accuracy. The two-step serial PCR reactions allow the specific and efficient amplification of targets, enabling our method to accommodate a wide range of original variant concentrations. This is critical, as the proportion of human DNA in stool ranged from undetectable to 9.929% in our study population (Supplementary Materials Table S3).

The detection rate of mutants in sDNA depends on several factors. First, the mutation detection rate depends on the rate in the local lesion. In our previous tissue study, we detected mutations in only 88% of CRC tumors and 60% of polyps using the Cancer Panel with 2855 COSMIC hotspots. Therefore, it is reasonable that we observed fewer mutants in stool when we detected only 21 target variants. Second, the mutation detection rate depends on the size and location of the neoplasm. Obviously, the larger the lesion, the more likely it will be to release mutant DNA into the stool. Ahlquist et al. [24] performed a case-control study using archived stool samples from 133 patients with adenomas ≥1 cm and found that the detection rate increased with adenoma size (54% ≤ 1 cm, 63% > 1 cm, 77% > 2 cm, 86% > 3 cm, and 92% > 4 cm). We observed the same phenomenon herein, as the detection rate was <5% when the lesion size was <1 cm but 40–44% in CRC with tumors > 3 cm (Figure 3D, Figure 4D). However, the location of the lesion did not seem to seriously impact the detection rate. Although it seems possible that the unprotected shed mutant DNA could be destroyed during its travel from the proximal intestine to the anus, we do not observe any significant correlation between the detection rate and the anatomical subsite (Figure 4C), which was consistent with a previous report [24]. Third, the mutation detection rate depends on the sensitivity of the method. Our sDNA panel examined 21 variants in five genes, opening the possibility for our results to be affected by the unequal performance of the variants in the multiplex extension reactions. Indeed, the LoVo cell line sensitivity test showed that KRAS p.G13D amplified better than APC p.R1114* (Figure 2). Our validation of stool samples from patients known to harbor the 20 expected mutations showed that only 10 of the mutations were successfully detected. In the future, therefore, it could be useful to seek ways to equalize the analytical sensitivity and/or increase the concentration of mutant DNA in stool. 

## 4. Materials and Methods

### 4.1. Selecting Genes and Variants for the sDNA Panel

In previous studies, we used NGS to obtain the mutated gene spectra for 53 formalin-fixed paraffin-embedded (FFPE) polyp samples and 50 fresh CRC tissue samples [10,11]. Here, we used our previous data to select the top five CRC-related genes (*APC*, *KRAS*, *TP53*, *PIK3CA*, and *SMAD4*) and identify 21 variants in these genes that were present in both polyps and tumor tissues, or in more than two patients with either polyps or tumors. We then used these variants to construct the sDNA panel (Table 1). Of the selected variants, most had high frequencies reported in the COSMIC database (≥1% [25]), whereas six were rare in the COSMIC database but frequent in our Taiwanese group.

### 4.2. LoVo Cell Line

The LoVo cell line was derived from a large intestinal carcinoma. Whole-exome screening identified a total of 3685 substitution/indel frameshift mutations in this cell line, which were recorded in the COSMIC database [25]. Among them, the APC p.R1114* and KRAS p.G13D mutations were identified in 44.6% of cell line DNA (in-house NGS data) and could be detected as a positive control in our sDNA panel. Therefore, to test the sensitivity of our developed assay, we subjected LoVo DNA to 2-fold serial dilution with wild-type human blood DNA, yielding 22.3%, 11.5%, 5.58%, 2.79% and 1.39% of mutant DNAs.

### 4.3. Assay Design and Study Subjects

To validate the analytical and clinical performance of the sDNA test, we use two cohorts of stool samples. Cohort 1 contained stool samples from 21 CRC patients, 13 patients with polyps, and nine normal controls. All of the included patients had known tissue mutations that we had previously identified by NGS of the Cancer Hotspot Panel [10,11]. We used this cohort to check the reliability of the assay by comparing the detection of mutants in paired tissue and stool samples (Figure 1A, cohort 1). For cohort 2, we prospectively enrolled 101 outpatients who presented at clinics for positive FIT results during 2014–2017. Upon colonoscopy, 20 of them were classified as normal, 27 had polyps, and 54 were diagnosed with CRC (Figure 1A, cohort 2).

Demographic features of the patients in the two cohorts are listed in Table 2. The classification of advanced adenoma included polyps with histological finding of tubular adenoma (largest diameter ≥1 cm), tubulovillous adenoma, and villous adenoma. The location of each lesion was classified as the right side (tumors at the cecum, ascending colon, hepatic flexure, and transverse colon), the left side (tumors at the splenic flexure, descending colon, and sigmoid colon), or the rectum. All patients provided written informed consent, and the study was approved by the Institutional Review Board of Chang Gung Memorial Hospital (101-4609A3; 102-5224B; 103-7047B).

### 4.4. Extraction of DNA from Stool Samples

Participants provided fresh stool samples (~1 g) before the day of colonoscopy or surgery. Stool samples (without any preservative) were frozen at −20 °C on the day of collection. Stool DNA was extracted using a QIAamp DNA Stool mini kit (QIAGEN, Germantown, MD, USA) according to the provided protocol, with a slight modification intended to enhance the final sDNA concentration. Briefly, 200 mg of stool was placed in a 2 mL tube, mixed with 1 mL Inhibit EX Buffer, thoroughly vortexed for 1 minute, heated for 5 minutes at 70 °C, and followed by centrifugation at 12,000 rpm for 1 min. The supernatant (400 μL) was transferred to a new Eppendorf tube containing 30 μL 20 mg/mL proteinase K, and then subjected to serial washing and a final elution of DNA with 60 μL of Buffer ATE. The DNA yield was quantified by calculating the A260/280 ratio using a NanoDrop Spectrophotometer (Thermo Fisher Scientific, Waltham, MA, USA).

### 4.5. RNaseP Real-time PCR for Quantitation of Human DNA

To identify the proportion of human DNA in the total stool DNA, we established an RNaseP real-time PCR method. Each PCR reaction volume of 20 μL included 5 μL stool DNA, 10 μL KAPA probe fast qPCR kit Master Mix (2×) Universal (KAPA Biosystems, Wilminton, MA, USA), 0.4 μL 50X TaqMan RNase P MGB assay set 4 (Applied Biosystems, Waltham, MA, USA), and 4.6 μL nuclease-free water. The PCR conditions consisted of 10 min at 95 °C followed by 40 cycles of 15 s at 95 °C and 1 min at 60 °C, as performed using a QuantStudio 12K Flex Real-Time PCR System (Thermo Fisher Scientific). The calibration curve for the amount of human DNA was constructed using 10-fold serially diluted human DNA obtained from blood (from 50 ng to 50 pg).

### 4.6. Establishment of a MassARRAY-based Method for Detecting Rare Mutations

The low proportion of mutation analysis was performed using the Sequenom MassARRAY platform (Agena Bioscience, San Diego, CA, USA). Briefly, the target region was PCR amplified from DNA with which contained a mixture of wild-type and variant DNA. A second round of PCR was then performed using an extension primer designed to anneal one nucleotide upstream of the mutated nucleotide, plus three of the four dideoxy nucleotide terminators (excluding the wild-type nucleotide). The terminated products were then separated with the unextension primer in matrix assisted laser desorption ionization-time of flight mass spectrometry (MALDI-TOF MS) and the variant type was identified based on the unique mass which incorporated into the last position of the amplicon. This method is called a ‘single allele base extension reaction’ (SABER) (Figure 1B). Theoretically, the MassARRAY SABER reaction can detect and analyze as little as 1% of target DNA against a large background of wild-type DNA [26].

In this study, we used the Assay Designer software package (v.4.0) to design 16 specific PCR primer pairs and 18 extension primer sequences to detect 21 variants in five CRC-related genes (Supplementary Materials Table S1). Extracted stool DNA (2 µL) was amplified in 35-µL multiplex PCR reactions containing 1.4 units of Taq polymerase, 2.5 pmol of each PCR primer, and 25 mM of each dNTP (PCR Accessory and Enzyme kit, Agena). Thermocycling was performed at 94 °C for 4 min followed by 45 cycles of 94 °C for 20 s, 56 °C for 30 s, and 72 °C for 1 min, and a final extension at 72 °C for 3 min. To avoid interference due to the similarity in the masses of the terminated products, each PCR product was divided among four reaction wells for the primer extension reaction and detection. Unincorporated dNTPs were deactivated using 0.3 U of shrimp alkaline phosphatase and SABER was initiated by adding the iPLEX Pro enzyme, SABER terminator mix, and extension primer mix. The reaction conditions consisted of 95 °C for 30 s, 40 cycles of 95 °C for 5 s plus five inner cycles of 52 °C for 5 s and 80 °C for 5 s, and a final extension at 72 °C for 3 min. A cation exchange resin was added to remove residual salt, and 7 nL of the purified primer extension product was loaded onto the matrix pad of a SpectroCHIP (Agena) and analyzed using a MassARRAY Analyzer 4. The mass detection window ranges from 4300 to 9000 Dalton and the areas of the mutation peak and unextended primer peak were acquired with the TYPER 4.0 software (Agena), and were used to calculate the extension ratio (ER) (Figure 1B). To prevent false-negative results caused by PCR inhibitors, an internal amplification control targeting a 150-bp region of the albumin gene was monitored for each sample. The threshold of each mutation point was defined as the mean plus 4SD of the wild-type ER value. Any sample with an ER value larger than the threshold was designated as being positive for the given mutation.

### 4.7. Statistical Analysis

Pearson’s Chi-square was used to calculate the significance of the between-group differences in the mutation detection rate. A *p*-value less than 0.05 (two-tailed) was considered statistically significant. To generate the risk-stratified score, we gave each variant a weighting factor. A FIT value > 500 ng/mL and any mutation in *APC* or *KRAS* was given a factor of 1 for each instance. Mutations in *TP53*, *PIK3CA*, and *SMAD4* were given a factor of 2 for each instance. The factors were summed to obtain a weighted score. Logistic regression and receiver operating characteristic curve analysis were used to evaluate the discriminatory power of this scoring system. All statistical analyses were conducted using SPSS Statistics 22 (SPSS Inc., Chicago, IL, USA).

## 5. Conclusions

In 2018, due to the rising incidence of CRC in younger individuals, the American Cancer Society (ACS) recommended that average-risk adults be screened for CRC beginning at age 45, which was 5 years earlier than the previous recommendation [27]. The guideline also recommended that positive results on screening tests should be followed up with timely colonoscopy. The implementation of this newly revised guideline will increase the demand for colonoscopy in FIT-positive individuals, meaning that physicians will need, more than ever before, objective data that may be used to prioritize patients for receipt of colonoscopy. In the present study, we introduce a simple and highly specific SABER MassARRAY system for detecting mutations in stool DNA. We calculated a risk-stratifying score, and found that there was a 4.538-fold increase in malignancy risk for each unit increase in the score. This score could thus serve as an effective means to guide patients and encourage them to adhere to subsequent surveillance. This work will contribute to patient management decisions and further cost-effectiveness analysis [28] in a larger cohort study with is warranted.

## Figures and Tables

**Table 1:** Contents of the CGMH CRC stool DNA panel (21 variants in 5 genes).

Gene	CDS Mutation	AA Mutation	CRC	Polyps	Mutation Frequency in COSMIC	Validated Sample ID (LOD%)
APC	c.2626C > T	p.R876*	3	2	2%	CRC-1445 (1.65%)
	c.3340C > T	p.R1114*	2	1	1%	LoVo cell line (1.4%)
	c.3871C > T	p.Q1291*	0	2	<1%	Polyp-44111 (3.64%)
	c.3916G > T	p.E1306*	0	2	1%	Polyp-36137 (2.8%)
	c.4348C > T	p.R1450*	0	4	5%	Polyp-13849 (26%)
	c.4661dupA	p.E1554fs	2	0	<1%	CRC-1480 (14.9%)
KRAS	c.34G > T/A	p.G12C/S	1	2	77%	CRC-1773 (2.4%)
	c.35G > T/A	p.G12V/D	12	8		CRC-1897(1.25%)
	c.38G > A	p.G13D	5	4	20%	LoVo cell line (1.4%)
	c.183A > C	p.Q61H	1	2	<1%	CRC-1897(1.75%)
	c.436G > A	p.A146T	2	1	<1%	CRC-1425(2.54%)
TP53	c.524G > A	p.R175H	4	0	11%	CRC-1445(2.7%)
	c.637C > T	p.R213*	3	0	2%	CRC-1738(4.87%)
PIK3CA	c.263G > A	p.R88Q	2	0	1%	CRC-1773(2.58%)
	c.1035T > A	p.N345K	2	0	<1%	CRC-1723(2.57%)
	c.1633G > A	p.E545K	4	0	31%	CRC-1480(3.04%)
	c.3073A > G/C	p.T1025A/P	2	0	<1%	CRC-1801(1.99%)
SMAD4	c.1082G > A	p.R361H	2	2	10%	CRC-1425(1.71%)

CDS: coding DNA sequence; AA: amino acid; CRC: colorectal cancer; COSMIC: Catalogue of Somatic Mutations In Cancer; *: stop codon; LOD: limit of detection.

**Table 2:** Clinical characteristics of the two patient cohorts.

Features	Cohort 1			Cohort 2		
	Normal	Polyps	CRC	Normal	Polyps	CRC
Sample no	9	13	21	20	27	54
Gender (M/F)	5/4	9/4	13/8	8/12	20/7	31/23
Age (mean ± SD)	64 ± 8	59 ± 7.6	68 ± 11.6	56.9 ± 8.9	65.3 ± 6.9	61.9 ± 11.1
FIT positive rate (%)	100%	46%	52%	100%	100%	100%
Lesion location						
Rectum		1	8		7	11
Left		7	9		16	21
Right		5	4		4	22
Lesion stage						
Benign polyps		3			19	
Advanced polyps		10			8	
Early stage of CRC (stage I-II)			11			27
Late stage of CRC (stage III-IV)			10			27
Lesion size (max diameter)						
<1 cm		6	0		22	0
1–3 cm		7	5		5	12
>3 cm		0	16		0	42

**Table 3:** Histological characteristics and mutations in paired tissue and stool samples from 13 polyps and 21 CRC patients.

Histological Classification	Sample ID	Sample Features				Expected Mutations by Stool Panel					Measured Mutations by Stool Panel					Concordance (Y/N)
		Stage	Location	Size-W (cm)	FIT	*APC*	*KRAS*	*TP53*	*PIK3CA*	*SMAD4*	*APC*	*KRAS*	*TP53*	*PIK3CA*	*SMAD4*	
**Adenoma**	91739	Tubular	Left	0.7	+											Y
	45248	Tubular	Left	1.2	−	p.R1114*										N
	88633	Tubular	Right	0.1	+	p.E1306*										N
	30523 ^2^	Tubular	Right	0.2	+											Y
	17766 ^3^	Tubulovillous	Left	0.9	+											Y
	90511	Tubulovillous	Left	1.1	−		p.Q61H									N
	18039	Tubulovillous	Left	2.2	+	p.R876*										N
	44111 ^3^	Tubulovillous	Right	1.4	−	p.Q1291*	p.G12V									N
	28808	Tubulovillous	Right	1.5	−		p.G12D									N
	29563	Villous	Rectum	2.2	−	p.R876*	p.G13D									N
	60570	Villous	Left	1.7	−	p.R1450*	p.G12S									N
	34335	Villous	Left	0.1	−		p.G12D/ p.G13D									N
	13849	Villous	Right	0.2	+	p.R1450*	p.G12V									N
**Early stage of CRC**	1325	II	Rectum	3.5	+			p.R213*								N
	1387	II	Rectum	3.5	−		p.G12V		p.E545K					p.E545K/ p.T1025A		Y
	1736	II	Rectum	3.8	+		p.G12V					p.G12V				Y
	1454	I	Left	2	−	p.R1114*					p.R1114*					Y
	1317	II	Left	2.3	+											Y
	1455	I	Left	3.1	−											Y
	1465	II	Left	3.5	+		p.G12V					p.G12V				Y
	1371	I	Left	4	+			p.R175H								N
	1423	II	Left	4.1	−		p.G12D									N
	1801	II	Right	2.8	+	p.R876*	p.G12V		p.T1025A					p.T1025A		Y
	1453	II	Right	8	−		p.G13D		p.E545K							N
**Late stage of CRC**	1897	III	Rectum	2.7	−		p.Q61H/ p.G12D									N
	1425	III	Rectum	4	+		p.A146T			p.R361H						N
	1657	III	Rectum	4.2	+		p.G12V									N
	1539	IV	Rectum	4.3	−											Y
	1738	III	Rectum	4.8	+			p.R213*					p.R213*			Y
	1840	III	Left	3.4	+		p.G12V	p.R175H				p.G12V	p.R175H			Y
	1480	III	Left	4.6	−	p.E1554fs	p.G12D		p.E545K					p.E545K		Y
	1773	III	Left	5	+		p.G12S		p.R88Q			p.G12S				Y
	1445	III	Right	2.3	−	p.R876*		p.R175H			p.R876*					Y
	1723	III	Right	3.9	−	p.R876*			p.N345K							N

Note 1: The superscript of the sample ID indicates the polyp number in the sample. Note 2: The red letter highlights the extra mutation found in stool in sample 1387.

## Supplementary Materials

The following are available online at https://www.mdpi.com/2072-6694/11/3/423/s1, Figure S1: Accuracy check of MassARRAY stool DNA panel, performed using diluted DNA from tissues with known mutations; Table S1: A. PCR primer sequences and amplicon sizes for the CGMH stool DNA panel. B. Extension primer sequences and their distributions in the four wells; Table S2: FIT value and variants detected using the stool DNA panel in 101 participants of cohort 2; Table S3: Proportion of human DNA in the tool DNA of cohort 1.
