# Supplementary material for: A Simple and Highly Specific MassARRAY-Based Stool DNA Assay to Prioritize Follow-up Decisions in Fecal Immunochemical Test-Positive Individuals
Source: Cancers (Basel). 2019 Mar 25;11(3):423. doi: 10.3390/cancers11030423 (PMC6468462; doi:10.3390/cancers11030423)

Supplementary Table 1.

A. PCR primer sequences and amplicon sizes for the CGMH stool DNA panel.

PCR Primer ID	Sequence	Amplicon size (bp)
APC_c.2626CtT-1st-PCRP	ACGTTGGATGTCTAGGCAACTACCATCCAG	117
APC_c.2626CtT-2nd-PCRP	ACGTTGGATGCATGACTTTGGCAATCTGGG	
APC_c.3340CtT-1st-PCRP	ACGTTGGATGTTTCTCCATACAGGTCACGG	117
APC_c.3340CtT-2nd-PCRP	ACGTTGGATGTGACACAAAGACTGGCTTAC	
APC_c.3871CtT-1st-PCRP	ACGTTGGATGTGCAGGGTATTAGCAGAATC	96
APC_c.3871CtT-2nd-PCRP	ACGTTGGATGCATCTTTGTCATCAGCTGAAG	
APC_c.G3916TF-1st-PCRP	ACGTTGGATGAGCTGACCTAGTTCCAATC	102
APC_c.G3916TF-2nd-PCRP	ACGTTGGATGAATCAGACGACACAGGAAGC	
APC_c.4348CtT-1st-PCRP	ACGTTGGATGCTCTCTCTTTTCAGCAGTAG	110
APC_c.4348CtT-2nd-PCRP	ACGTTGGATGAGAAGTAAAACACCTCCACC	
APC_c.4660-4661-1st-PCRP	ACGTTGGATGCTGAATCATCTAATAGGTCC	104
APC_c.4660-4661-2nd-PCRP	ACGTTGGATGTCAGAGCAGCCTAAAGAATC	
KRAS_c.34-38 exon2-1st-PCRP	ACGTTGGATGAGGCCTGCTGAAAATGACTG	100
KRAS_c.34-38 exon2-2nd-PCRP	ACGTTGGATGTTAGCTGTATCGTCAAGGCA	
KRAS_c.A183 exon3-1st-PCRP	ACGTTGGATGATGTACTGGTCCCTCATTGC	97
KRAS_c.A183 exon3-2nd-PCRP	ACGTTGGATGGATTCCTACAGGAAGCAAGTA	
KRAS_c.G436AF exon4-1st-PCRP	ACGTTGGATGTTCAGTGTTACTTACCTGTC	101
KRAS_c.G436AF exon4-2nd-PCRP	ACGTTGGATGGGCTCAGGACTTAGCAAGAA	
TP53_c.524G>AR-1st-PCRP	ACGTTGGATGTCTACAAGCAGTCACAGCAC	102
TP53_c.524G>AR-2nd-PCRP	ACGTTGGATGTGCTCACCATCGCTATCTGA	
TP53_c.637C>T-1st-PCRP	ACGTTGGATGAGTTGCAAACCAGACCTCAG	105
TP53_c.637C>T-2nd-PCRP	ACGTTGGATGGCGTGTGGAGTATTTGGATG	
PIK3CA_c.G263-333-1st-PCRP	ACGTTGGATGGAAAGGGACAACAGTTAAGC	102
PIK3CA_c.G263-333-2nd-PCRP	ACGTTGGATGCGTAAGTGTTACTCAAGAAGC	
PIK3CA_c.1035T>A-1st-PCRP	ACGTTGGATGGCATCAGCATTTGACTTTACC	117
PIK3CA_c.1035T>A-2nd-PCRP	ACGTTGGATGGGGTTATAAATAGTGCACTC	
PIK3CA_c.G1633A-1st-PCRP	ACGTTGGATGTAGCACTTACCTGTGACTCC	91
PIK3CA_c.G1633A-2nd-PCRP	ACGTTGGATGTACACGAGATCCTCTCTCTG	
PIK3CA_c.A3073GF-1st-PCRP	ACGTTGGATGTACTCCAAAGCCTCTTGCTC	114
PIK3CA_c.A3073GF-2nd-PCRP	ACGTTGGATGCTCTGGAATGCCAGAACTAC	
SMAD4_c.1081-1082-1st-PCRP	ACGTTGGATGCTGTGGACATTGGAGAGTTG	90
SMAD4_c.1081-1082-2nd-PCRP	ACGTTGGATGCTGTTGATGGATACGTGGAC	

B. Extension primer sequences and their distributions in the four wells.

**Extension Primer ID**	**Sequence**	**Well ID**
APC_c.C2626T	GGAGATCTGCAAACCTC	4
APC_c.C3340T	GCCAATGGTTCAGAAACAAAT	2
APC_c.C3871T	TGAAGATGAAATAGGATGTAAT	2
APC_c.G3916T	CTAATACCCTGCAAATAGCA	4
APC_c.C4348T	CTCAAACAGCTCAAACCAAG	2
APC_c.4661dupA	CCTTTTCAGAATCAATAGTTTTTTT	4
KRAS_c.G34ToAF	TaGTGGTAGTaGGAGCT	1
KRAS_c.G35AoTR	CACTCTTGCCTACGCCA	2
KRAS_c.G38AF	AGGCACTCTTGCCTACG	2
KRAS_c.A183CF	gCTCGACACAGCAGGTCA	3
KRAS_c.G436AF	AATTCCTTTTATTGAAACATCA	1
TP53_c.G524AR	GCTCATGGTGGGGGCAG	4
TP53_c.C637T	GGATGACAGAAACACTTTT	2
PIK3CA_c.G263AF	AGCCGAAGGTCACAAAGT	4
PIK3CA_c.T1035A	CTTATCAATGTCTCGAATATTTAC	3
PIK3CA_c.G1633A	ccAGAAAATCTTTCTCCTGCT	4
PIK3CA_c.A3073GF	TGACATTGCATACATTCGAAAG	3
SMAD4_c.G1082AF	CGTGGACCCTTCTGGAGGAGATC	1

Supplementary Table 2. FIT value and variants detected using the CGMH stool DNA panel in 101 participants of cohort 2.

Stool ID	Age	Gender (F=0, M=1)	Diagnosis	FIT (ng/mL)	Presence of stool variant					Sum of score (FIT+ variants)
					APC	KRAS	TP53	PIK3CA	SMAD4	
				Weighting factor=1 when >=500	Weighting factor=1	Weighting factor=1	Weighting factor=2	Weighting factor=2	Weighting factor=2	
372	53	0	Normal	121						0
798	58	1	Normal	137						0
819	47	1	Normal	100						0
820	53	0	Normal	319						0
821	34	1	Normal	100						0
822	63	1	Normal	215						0
823	67	0	Normal	404						0
851	59	0	Normal	100						0
852	58	0	Normal	465						0
855	53	1	Normal	138						0
856	75	0	Normal	305						0
866	53	0	Normal	134						0
958	62	1	Normal	103						0
1151	52	1	Normal	345						0
1152	58	0	Normal	100						0
1158	53	0	Normal	286						0
384	52	1	Normal	780						1
813	73	0	Normal	>1000						1
875	58	0	Normal	>1000						1
2798	56	0	Normal	928						1
288	61	1	B-polyp	222						0
332	54	1	B-polyp	328						0
792	74	1	B-polyp	133						0
799	67	0	B-polyp	113						0
803	74	0	B-polyp	410						0
805	61	0	B-polyp	432						0
812	55	1	B-polyp	162						0
859	74	1	B-polyp	149						0
953	55	1	B-polyp	194						0

1112	60	0	B-polyp	100						0
1183	66	1	B-polyp	299						0
2807	62	1	B-polyp	165						0
836	72	1	B-polyp	308						0
863	73	1	B-polyp	>1000						1
1156	78	1	B-polyp	>1000						1
2806	60	1	B-polyp	784						1
2901	72	1	B-polyp	644						1
816	72	1	B-polyp	260			p.R175H			2
381	59	0	B-polyp	723		p.G12A				2
796	66	1	Ad-polyp	372						0
957	73	0	Ad-polyp	119						0
1113	67	1	Ad-polyp	345						0
1157	60	1	Ad-polyp	134						0
2792	59	0	Ad-polyp	125						0
959	65	1	Ad-polyp	>1000						1
2805	61	1	Ad-polyp	>1000						1
2795	64	1	Ad-polyp	520	p.R1114*	p.G12D				3
775	70	1	Early-CRC	428						0
830	81	1	Early-CRC	418						0
1184	50	0	Early-CRC	278						0
1556	50	0	Early-CRC	390						0
1665	63	0	Early-CRC	180						0
776	70	0	Early-CRC	>1000						1
872	64	1	Early-CRC	>1000						1
1162	59	0	Early-CRC	808						1
1181	66	0	Early-CRC	>1000						1
1186	73	1	Early-CRC	>1000						1
1281	60	1	Early-CRC	>1000						1
1523	48	1	Early-CRC	>1000						1
1525	50	1	Early-CRC	>1000						1
1580	60	0	Early-CRC	>1000						1
1582	48	0	Early-CRC	>1000						1
1591	61	1	Early-CRC	>1000						1
1610	58	1	Early-CRC	>1000						1
1615	79	1	Early-CRC	609						1
769	45	1	Early-CRC	>1000		p.G13D				2

811	65	1	Early-CRC	>1000		p.G12D				2
848	51	1	Early-CRC	>1000		p.G13D				2
1114	62	0	Early-CRC	>1000		p.G12D				2
1283	59	1	Early-CRC	>1000		p.G13D				2
1577	64	1	Early-CRC	>1000		p.G12D				2
774	71	0	Early-CRC	895			p.R175H			3
841	60	1	Early-CRC	>1000			p.R175H			3
788	61	0	Early-CRC	>1000		p.G12V		p.E545K		4
784	55	1	Late-CRC	248						0
802	74	0	Late-CRC	324						0
1174	57	1	Late-CRC	126						0
1277	74	1	Late-CRC	474						0
1570	42	0	Late-CRC	230						0
791	74	0	Late-CRC	>1000						1
829	65	0	Late-CRC	>1000						1
1175	73	0	Late-CRC	714						1
1179	57	0	Late-CRC	>1000						1
1284	76	1	Late-CRC	>1000						1
1302	70	1	Late-CRC	>1000						1
1522	77	0	Late-CRC	>1000						1
1555	64	1	Late-CRC	>1000						1
1598	73	0	Late-CRC	>1000						1
1648	61	1	Late-CRC	>1000						1
826	54	1	Late-CRC	190			p.R175H			2
846	64	1	Late-CRC	456	p.R1114*	p.G12D				2
867	85	0	Late-CRC	>1000		p.G12A				2
1150	49	1	Late-CRC	>1000		p.G12D				2
1163	61	1	Late-CRC	>1000	p.R876*					2
1187	63	0	Late-CRC	>1000		p.G12V				2
779	51	1	Late-CRC	>1000	p.R876*	p.G12D				3
1301	22	1	Late-CRC	>1000	p.R1450*	p.G12D				3
1581	63	1	Late-CRC	100		p.A146T		p.E545K		3
1644	63	0	Late-CRC	>1000	p.R876*	p.A146T				3
1185	66	1	Late-CRC	>1000	p.R1450*	p.A146T	p.R175H			5
1153	60	0	Late-CRC	>1000	p.R1450*<br>p.R876*	p.G12V	p.R175H	p.E545K		7

Supplementary Table 3. Proportion of human DNA in the CGMH stool DNA of cohort 1.

Group	Sample ID	Stool DNA conc. (ng/uL)	Human DNA calculated by RNase P PCR (ng/uL)	Human DNA in total stool DNA (%)
Normal	N-1	109.9	6.66	6.056
	N-2	1156.8	0.14	0.012
	N-3	591.2	0.20	0.034
	N-4	278.5	3.09	1.109
	N-5	681.1	2.57	0.377
	N-6	347.9	0.84	0.241
	N-7	1291.6	0.07	0.005
	N-8	598.9	0.17	0.029
	N-9	1772	0.24	0.013
Polyps	45605	955.6	0.04	0.004
	36137	65.3	0.78	1.200
	91739	914.8	0.06	0.006
	45248	665	0.24	0.036
	88633	190.8	2.77	1.454
	30523	70	0.85	1.220
	17766	1668.3	0.11	0.007
	90511	924.9	0.44	0.047
	18039	195.2	0.90	0.460
	44111	494.4	0.35	0.070
	28808	1894.1	0.01	0.000
	60570	396.9	23.71	5.973
	34335	802.4	0.22	0.028
	13849	270.3	2.77	1.026
	29563	271.8	0.19	0.070
CRC	1325	69.9	3.22	4.613
	1387	180.8	0.03	0.019
	1736	37	0.62	1.684

1454	72.4	0.33	0.454
1317	13.5	0.35	2.568
1455	83.9	4.30	5.122
1465	66.4	0.83	1.252
1371	230.6	6.45	2.796
1423	484.7	0.09	0.018
1801	692.1	0.62	0.090
1453	421.6	0.17	0.039
1897	652.5	1.09	0.167
1425	20.9	2.08	9.929
1657	638.6	0.61	0.095
1539	492.3	0.02	0.004
1738	116.7	3.14	2.693
1840	109.2	0.98	0.895
1480	272.5	0.24	0.088
1773	307	0.24	0.080
1445	64.1	2.82	4.398
1723	31.6	0.54	1.722

Supplementary figure. Accuracy check of MassARRAY stool DNA panel, performed using diluted DNA from tissues with known mutations

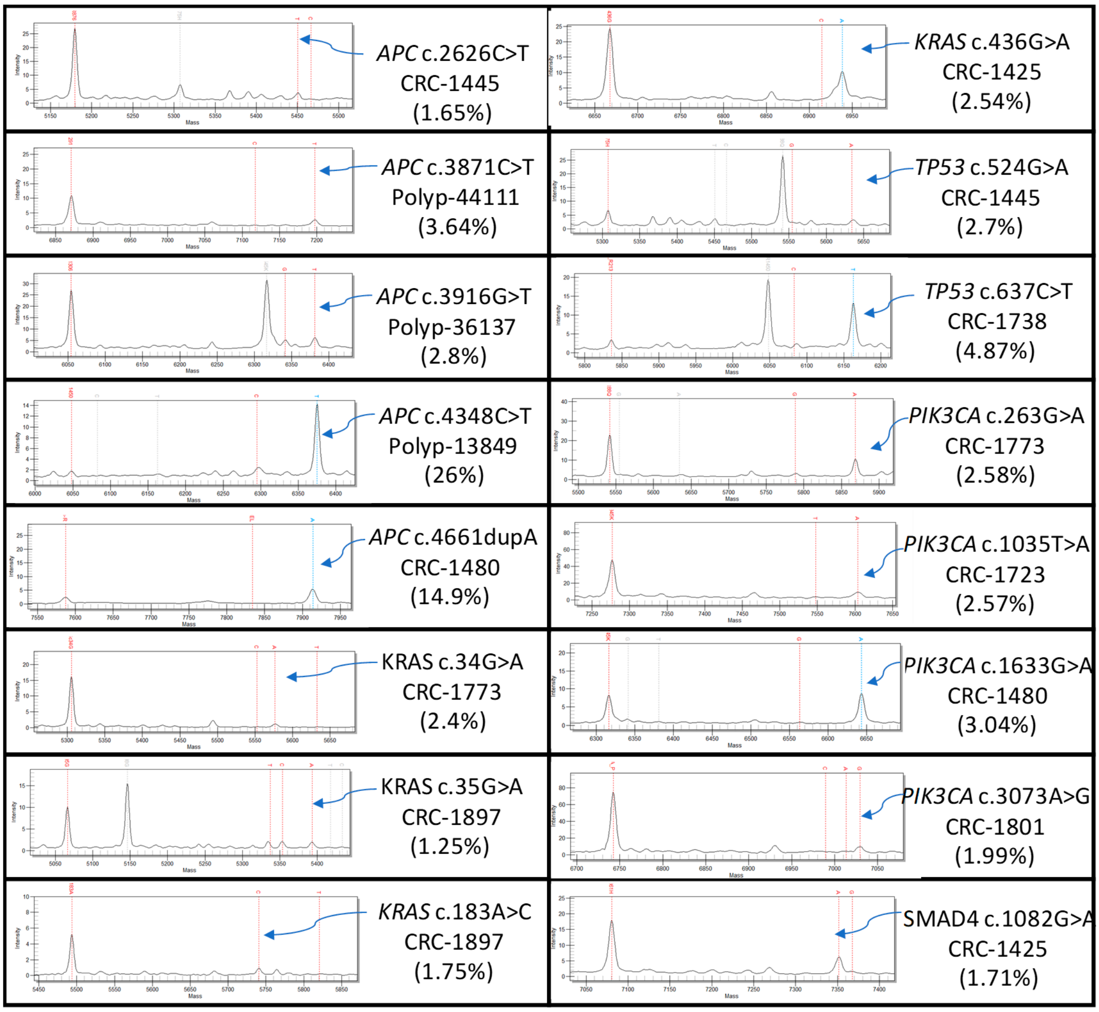

Supplement: Supplementary file 1 [file cancers-11-00423-s001.pdf]
